# Personalized Strategies for Head and Neck Reconstruction Using Pedicled Flaps

**DOI:** 10.3390/jpm16020112

**Published:** 2026-02-13

**Authors:** Giuseppe Riva, Andrea Canale, Gian Marco Motatto, Virginia Talamelli, Marco Briguglio, Alice Bordin, Teodros Veronesi, Giancarlo Pecorari

**Affiliations:** 1 Division of Otorhinolaryngology, Department of Surgical Sciences, University of Turin, Via Genova 3, 10126 Turin, Italy; griva@cittadellasalute.to.it (G.R.); gmotatto@cittadellasalute.to.it (G.M.M.); virginia.talamelli@unito.it (V.T.); alicebordin0896@gmail.com (A.B.); teodros.veronesi@edu.unito.it (T.V.); giancarlo.pecorari@unito.it (G.P.); 2 Division of Otorhinolaryngology, Chivasso Hospital, Via Paolo Regis 42, 10034 Chivasso, Italy; marco.briguglio.1991@gmail.com

**Keywords:** pedicled flap, head and neck cancer, reconstruction, necrosis

## Abstract

**Background/Objectives:** In recent decades, free flaps have emerged as the gold standard for head and neck reconstruction. However, their use is contraindicated in some cases due to advanced age and/or comorbidities. In such patients, a pedicled flap may be considered. The aim of this observational study was to evaluate strategies for head and neck reconstruction using pedicled flaps in the era of free flaps. Furthermore, the complication rate was analyzed. **Methods:** Patients who underwent head and neck reconstruction with pedicled flaps were included. The following flaps were considered: the pectoralis major (PMF), deltopectoral, platysma, frontal, temporal, nasolabial, supraclavicular artery island (SCAIF), infrahyoid, sternocleidomastoid, buccal fat pad, and facial artery myomucosal flap (FAMM). Patients’ characteristics, flap type, recipient sites, and flap-related complications were systematically recorded. **Results:** A total of 112 pedicled flaps were analyzed. A PMF was most commonly used for tongue and hypopharyngeal reconstruction. Partial and complete flap necrosis occurred in 11.6% and 1.8% of cases, respectively. Wound dehiscence was reported in 12.5% of cases, while pharyngo-/oro-cutaneous fistulas developed in 6.3% of patients. Hemorrhage from the donor site or flap occurred in 3.6% of cases, and pharyngeal stenosis in 0.9%. **Conclusions:** Each reconstructive strategy depends on the site and extent of tissue loss. Given the low complication rates, pedicled flaps remain a valid option for head and neck reconstruction in selected patients.

## 1. Introduction

Head and neck oncologic resections frequently result in complex three-dimensional defects that require meticulous reconstructive planning to restore function—speech, swallowing, and airway patency—as well as aesthetics. Over the past four decades, microvascular free-flap surgery has become a mainstay due to its versatility, reliable vascularity, and capacity to address composite tissue deficits with high success rates [1,2,3]. Nevertheless, not all patients are ideal candidates for free flaps. Patient factors—advanced age, cardiopulmonary disease, diabetes, renal insufficiency, prior radiotherapy, recurrent disease—or poor vascular status can render microvascular procedures risky, prolonged, or impractical [3,4,5]. As treatment is increasingly individualized to tumor biology and patient comorbidity, reconstructive choices should integrate defect geometry (site, size, and depth), prior treatments, expected adjuvant therapy, functional priorities, frailty, and social context. System-level considerations also matter: operating room availability, postoperative monitoring resources, and microsurgical expertise influence real-world decision-making and may limit access to free tissue transfer, especially in salvage or palliative settings [4,6]. In addition, when they are carefully selected, pedicled flaps can achieve complication rates and functional results comparable to free flaps in high-risk cohorts, with very low total flap loss and largely manageable minor complications [6].

Within such an individualized algorithm, pedicled flaps are not inferior or outdated, but are context-driven solutions that complement free flaps across a flexible reconstructive spectrum. Their advantages include shorter operative duration, avoidance of microvascular anastomosis, reduced reliance on intensive monitoring, and often earlier resumption of adjuvant therapies—an oncologic priority [1,2].

In confirmation of these data, the recent literature has reappraised pedicled flaps within modern reconstructive pathways: the supraclavicular artery island flap (SCAIF) has gained traction for intraoral and pharyngeal resurfacing owing to its thin, pliable paddle and low donor morbidity [7,8,9]; the pectoralis major flap (PMF)—often myofascial—remains indispensable for pharyngeal closure after salvage laryngectomy and for vascular coverage in vessel-depleted necks [10,11]; and mucosal and local options such as the facial artery myomucosal flap (FAMM), buccal fat pad (BFP), infrahyoid flap (IHF), and platysma provide elegant solutions for small-to-medium intraoral defects with favorable functional outcomes [12,13,14,15].

The aim of this observational study was to evaluate strategies for head and neck reconstruction using pedicled flaps in the era of free flaps in order to identify a personalized approach for each patient. Furthermore, the complication rate was analyzed.

## 2. Materials and Methods

Patients who underwent head and neck reconstruction with pedicled flaps between 2017 and 2025 at our department were included. The following flaps were considered: the pectoralis major (PMF), deltopectoral (DP), platysma, paramedian forehead (PFF), temporal, nasolabial, supraclavicular artery island (SCAIF), infrahyoid (IHF), sternocleidomastoid (SCM), buccal fat pad (BFP), and facial artery myomucosal flap (FAMM). A pedicled flap was chosen due to advanced age, comorbidities, and/or previous treatments (surgery and/or radiotherapy) that contraindicated a free flap, or the patient’s preference.

Exclusion criteria included an age < 18 years, the use of pedicled flaps for reconstruction of areas outside the head and neck, and the use of small transposition/rotation cutaneous flaps. All procedures were performed in accordance with the ethical standards of the institutional research committee and the 1964 Helsinki Declaration and its amendments or equivalent ethical guidelines. Written informed consent was obtained from all patients. Approval was granted by the Institutional Review Board (A.O.U. Città della Salute e della Scienza di Torino—A.O. Ordine Mauriziano—A.S.L. Città di Torino). According to the study protocol, retrospective data collection was performed for patients treated between January 2017 and January 2023, while data of patients who underwent surgery between February 2023 and June 2025 were prospectively collected.

Patient characteristics, flap types, recipient sites, and flap-related complications were systematically recorded. Specifically, the following postoperative complications were analyzed: partial and complete necrosis, wound dehiscence at the recipient site, fistula, bleeding, and pharyngeal stenosis.

Statistical analysis was conducted using the Statistical Package for the Social Sciences (SPSS), version 26.0. As the Kolmogorov–Smirnov test indicated a non-Gaussian distribution of variables, non-parametric tests were applied. Descriptive analysis was performed, with data presented as medians and interquartile ranges (IQR) or as percentages. The Chi-square or Fisher’s exact test was used for categorical variables. A *p*-value below 0.05 was considered statistically significant.

## 3. Results

This study included 112 pedicled flaps performed in 108 patients for head and neck reconstruction. Flap elevation was performed concomitantly with tumor resection in 93 cases, while 19 cases were related to traumatic injuries (*n* = 1) or postoperative complications, such as pharyngo- and oro-cutaneous fistulas (*n* = 13), and wound dehiscence or necrosis of a previous flap (*n* = 5).

The median age was 69.5 years (IQR 19; range 25–95 years). The majority of patients were male (*n* = 77, 71.3%) and smokers (*n* = 91, 84.2%). Table 1 provides a summary of the causes of tissue deficit, surgical interventions, and recipient sites.

**Figure 1:**
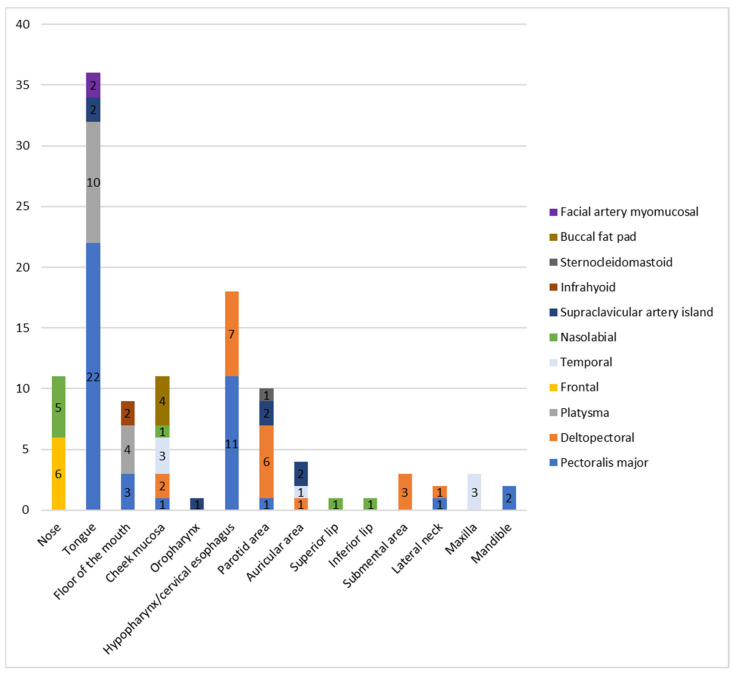
Pedicled flaps (*n*) used to reconstruct each recipient site.

**Figure 2:**
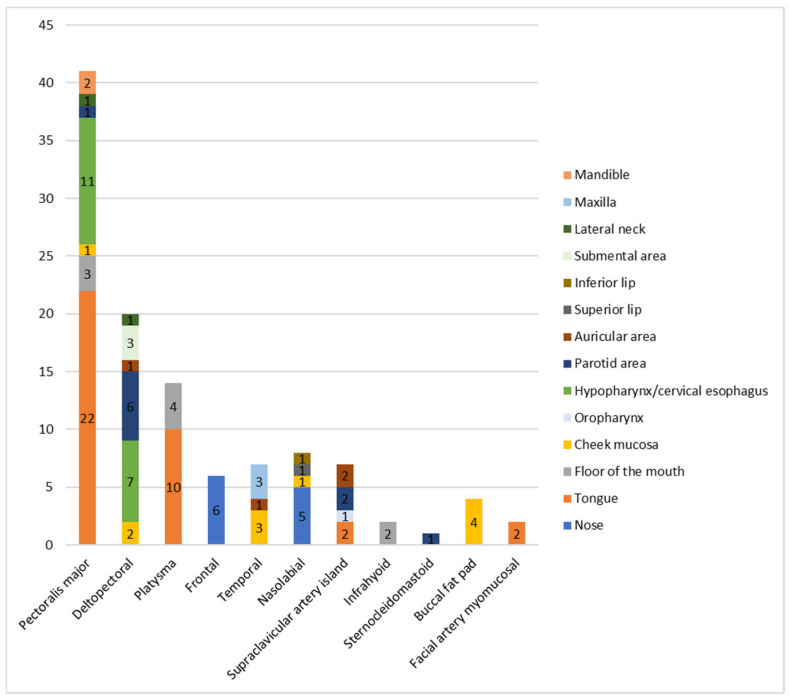
Recipient sites (*n*) reconstructed with each pedicled flap.

Figure 1 illustrates the distribution of pedicled flaps across recipient sites, while Figure 2 shows the recipient sites reconstructed with each flap. The PMF was harvested as a myocutaneous flap in 14 of 41 cases and as a myofascial flap in the remaining 27 cases.

**Figure 3:**
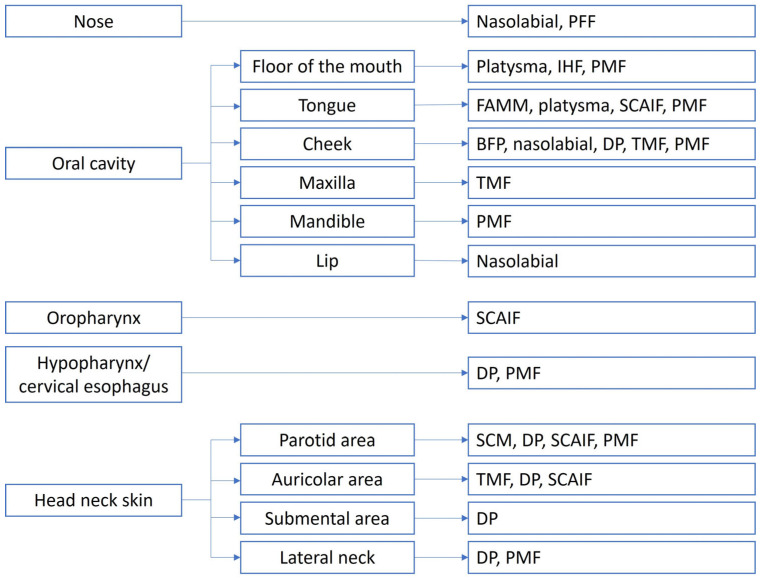
Algorithm for tailored and personalized reconstruction in the head and neck. For each site, the reconstructive strategies with pedicled flaps in order of extent of tissue deficit are highlighted. BFP, buccal fat pad flap; DP, deltopectoral flap; FAMM, facial artery myomucosal flap; IHF, infrahyoid flap; PFF, paramedian forehead flap; PMF, pectoralis major flap; SCAIF, supraclavicular artery island flap; SCM, sternocleidomastoid muscle flap; TMF, temporal muscle flap.

**Figure 4:**
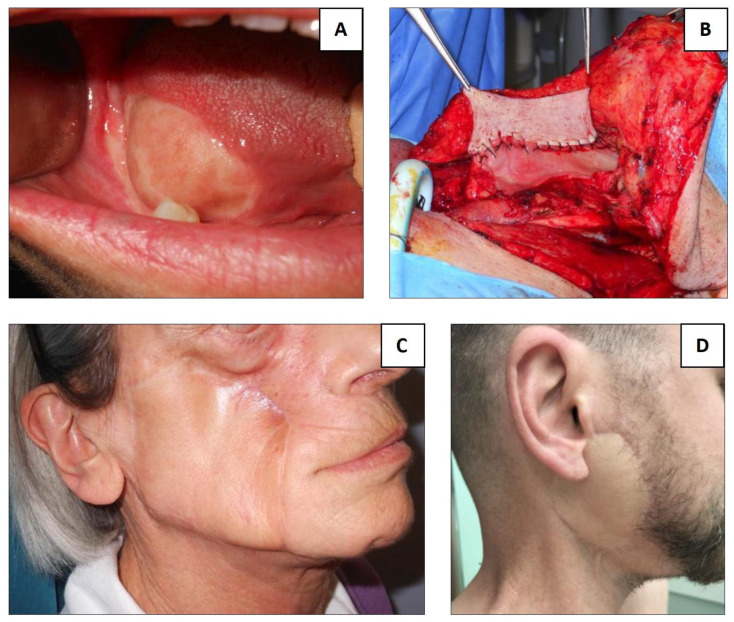
Clinical cases: (**A**) platysma flap for tongue reconstruction after partial glossectomy; (**B**) PMF for hypopharyngeal reconstruction after total laryngectomy with resection of one piriform sinus; (**C**) DP flap for cheek skin reconstruction; (**D**) SCAIF for reconstruction of the parotid area after total parotidectomy with skin resection.

For each reconstruction site, flap selection was based on prior surgical procedures and the extent of tissue deficit, both in width and depth. The portion of the flap intended for reconstruction was required to match the width and thickness of the defect without undue tension. An algorithm for individualized reconstruction is presented in Figure 3, while Figure 4 highlights some clinical cases.

**Figure 5:**
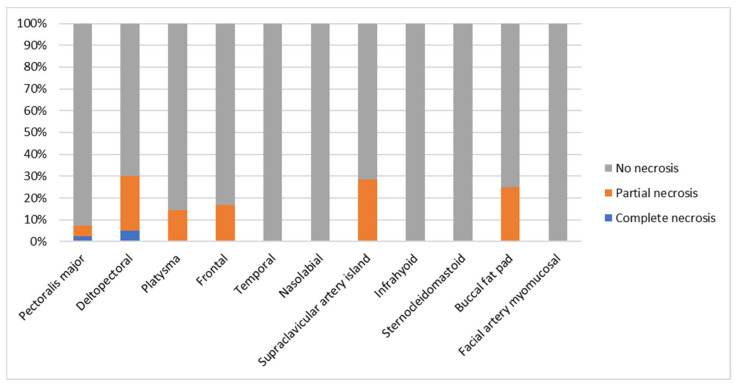
Partial and complete necrosis for each pedicled flap.

Complications were observed in 32 flaps (28.6%). Partial and complete flap necrosis occurred in 13 (11.6%) and 2 (1.8%) cases, respectively. Wound dehiscence was noted in 14 cases (12.5%), while pharyngo-/oro-cutaneous fistulas developed in 7 (6.3%). Hemorrhage from the donor site or the flap was reported in four cases (3.6%), and pharyngeal stenosis in one case (0.9%). Although differences among flap types were not statistically significant (*p* > 0.05), the highest complication rate was observed in the deltopectoral flap group (Table 2, Figure 5).

Most complications—including partial necrosis, wound dehiscence without fistula, bleeding, and small fistulas—were managed conservatively with local treatments and/or dressings (e.g., secondary intention healing, suturing under local anesthesia, or debridement and closure of minor fistulas). In contrast, complete necrosis or large fistulas required reconstruction with a new pedicled flap (PMF).

## 4. Discussion

Pedicled flaps remain an essential element of contemporary head and neck reconstruction, complementing free tissue transfer when patient- or system-level constraints preclude lengthy microsurgery, in salvage and palliative contexts, or when regional tissue is simply the best match for a three-dimensional defect [1,2,3]. Our series shows that a tailored menu of pedicled options can address nasal, oral, pharyngeal, auricular, and cervicofacial defects while maintaining acceptable complication profiles, aligning reconstructive choice with the realities of multimorbid, irradiated, or vessel-depleted patients. Each head and neck subsite was evaluated for a personalized solution.

− Nose

For nasal skin and lining, the PFF was favored for multi-subunit or large composite defects due to its robust axial perfusion and staged thinning potential, while nasolabial flaps were used for smaller peri-alar/alar losses that benefit from a color–texture match and single-stage transposition. Contemporary cohorts reaffirm PFF reliability and the importance of risk-factor optimization (e.g., smoking) to minimize wound issues, whereas nasolabial flaps provide dependable resurfacing with minimal donor morbidity in limited defects [16,17].

− Tongue

In our experience, the PMF was the predominant choice for tongue reconstruction, particularly when bulk, vascular protection, or dead-space obliteration were priorities (e.g., prior irradiation or vessel-depleted necks), with functional outcomes consistent with modern practice [10,11]. For mucosal defects requiring a thin and pliable lining without bulk, we made extensive use of the platysma flap; the FAMM flap was employed only in edentulous patients or in those missing at least the posterior dentition, to preserve the arc of rotation and avoid pedicle trauma [12,14,18,19,20].

− Floor of the mouth

We relied on the IHF, the platysma flap, and the PMF, chosen according to reach, required thickness, and the history of neck surgery/irradiation. The IHF offers thin, reliable tissue with favorable pooled outcomes; the platysma provides pliable coverage when designed within safe length-to-width ratios [14,15,18].

− Cheek mucosa

Cheek lining defects could be reconstructed with multiple affordable techniques: nasolabial, DP, temporal and PMF when a larger surface area was required. The BFP assists shallow posterior defects with predictable epithelialization. This mirrors contemporary algorithms that leverage mucosa-like pliability for intraoral function and fasciocutaneous paddles when circumferential reach or skin replacement is needed [12,13,14,15,18,21].

− Oropharynx

For resurfacing the tonsillar fossa and posterior pharyngeal wall in patients unfit for microsurgery, the SCAIF provided thin, pliable lining with a reliable pedicle and low donor-site morbidity, provided that tunneling was gentle, the pedicle was de-epithelialized and its kinking avoided, and the mucosal closure remained tension-free [7,8,9].

− Hypopharynx/cervical esophagus

After laryngopharyngectomy—especially in previously irradiated necks—the PMF (often myofascial) was used to augment or patch the neopharynx and to protect against fistula or repair existing ones, while DP was considered when a small fistula was present or the PMF was contraindicated. In the literature, a combined approach is described for selected circumferential or near-circumferential reconstructions in patients unsuitable for free tissue transfer, involving a posterior wall DP segment with an anterior PMF or SCAIF component [10,11,22,23,24,25].

− Parotid and auricular area

While the temporal flap is a valid, thin, well-vascularized option reported in other series, in our practice the SCAIF and DP were the most frequently used, and provided reliable coverage with an acceptable contour and a low rate of complications [26]. Evidence supports the SCM for reconstruction and potential mitigation of Frey syndrome with low morbidity as well [7,8,25,27].

− Upper and lower lips

Superiorly or inferiorly based nasolabial flaps provided dependable cutaneous and mucosal replacement for partial-thickness lip defects with good symmetry and oral competence. Where mucosal lining adjacent to the vermilion is required without bulk, the FAMM flap may be used [12,28].

− Submental area

In all three cases in our series, we used the DP flap for submental/anterior neck coverage, capitalizing on its broad skin paddle and straightforward execution for medium-to-large cervical defects, with results in line with recent experiences using the DP for the cervical/submental region [25,29].

− Lateral neck and vessel coverage

In vessel-depleted or previously dissected/irradiated necks, the PMF was chosen for robust protection of carotid and jugular structures and for dead-space obliteration, whereas the DP provided thinner resurfacing without bulk [23,25].

− Maxilla/palatal interface

Following limited maxillectomy, the BFP resurfaced palatal defects due to proximity, ease of harvest, and predictable epithelialization; for larger midface defects in patients unfit for osseous free transfer, temporalis-based reconstruction remained a salvage pathway with acceptable morbidity and contour [13,21].

− Mandible (soft-tissue lining/plate coverage)

When osseous reconstruction was not pursued or was contraindicated, the PMF provided dependable bulk to reline the oral cavity, protect plates or exposed bone, and facilitate timely adjuvant therapy, consistent with contemporary institutional experiences [30]. In this study, we reported two cases of lateral segmental mandibulectomy that underwent reconstruction with plates and the PMF with intraoperative establishment of occlusion. We did not have any plate exposure. However, it is important to consider that plate coverage by the PMF might not work when the defect is in the anterior mandible and the plate may migrate through again. Moreover, adjuvant radiotherapy may cause plate exposure.

− Complications

Benchmarking our overall complication profile (partial necrosis 11.6%, complete necrosis 1.8%, wound dehiscence 12.5%, pharyngo-/oro-cutaneous fistula 6.3%, bleeding 3.6%, and pharyngeal stenosis 0.9%) against the literature shows broad concordance with modern pedicled-flap practice. For the SCAIF, contemporary appraisals report overall complications around 17% with partial flap loss ≈5–6% and pharyngeal leak ≈3% [7,8,9]. Where partial-necrosis risk is a concern, intraoperative indocyanine green (ICG) fluorescence video-angiography can help mitigate distal-tip compromise by mapping the supraclavicular axis, checking perfusion after tunneling/rotation, and allowing selective distal trimming of hypoperfused segments before inset; adoption depends on device availability and cost, which are not universal [31,32,33,34]. For infrahyoid flaps, pooled meta-analytic estimates (partial loss 10.4%, total loss 1.8%, and fistula 3.0%) mirror our global figures, supporting their reliability for oral cavity/oropharyngeal reconstruction when properly selected [15]. For pectoralis major variants, modern analyses confirm rare total loss with variability of partial loss depending on design and salvage indications; our PMF outcomes are in line with these trends [10,11]. Representative series report overall complications around 12.3% (major, 3.5%, and minor, 8.8%) with no total or partial flap loss in 114 PMF cases [35]. Finally, because the deltopectoral (DP) flap carried one of the highest complication burdens in our cohort, it is notable that recent series report aggregated distal necrosis around ~20–30% of cases, driven by distal-tip ischemia beyond the second angiosome; our findings fall within that range [25]. Literature-supported measures to mitigate DP necrosis include respecting length-to-width ratios, employing delay when planning long paddles or tubed segments, avoiding excessive lateral extension, using intraoperative indocyanine green (ICG) fluorescence video-angiography, and achieving a tension-free inset; carefully selected single-stage approaches for cervical defects can also perform well when perfusion principles are respected [25,32].

− Study limitations

This retrospective single-center analysis included small sample sizes for several reconstructed subsites (e.g., oropharynx, upper and lower lip, mandible, and lateral neck), which limited the precision of site-specific estimates and the power to detect differences among flap types. Further studies must include a comparison of complication rates and outcomes among different flaps stratifying for defect location and sublocalization within the head and neck region. Selection bias is inherent to a pedicled-flap cohort (often multimorbid and heavily pretreated), and functional outcomes were not formally quantified across all domains. Long-term functional and aesthetic outcomes should be included in future studies. Furthermore, the submental island flap was missing in our case series. It is a workhorse flap in head and neck oncology and should be included in further studies. Finally, comparison with a free-flap cohort was lacking, as were stratifying by localization/sublocalization and reporting differences in complications, outcomes, length of hospital stay, and other clinically relevant endpoints. Future research should include a larger, preferably multicenter, series with harmonized reconstructive algorithms to validate these personalized, site-based recommendations.

## 5. Conclusions

In the free-flap era, pedicled flaps remain essential tools for oncologic head and neck reconstruction, particularly in elderly, comorbid, irradiated, or salvage patients, and in resource-constrained settings. Across 112 pedicled flaps, our overall complication burden was modest: complete loss was rare (1.8%), most events were minor and conservatively managed, and fistula/stenosis rates were within the lower ranges reported for similar high-risk cohorts. A site- and extent-based strategy—leveraging the forehead flap for complex nasal defects; FAMM/BFP flaps for small oral mucosal losses; platysma and IHF flaps for broader intraoral resurfacing; the PMF for wide tongue defects, hypopharyngeal closure, vessel coverage, and high-risk laryngectomy reinforcement; SCAIF and DP flaps for superficial skin losses (noting that the DP flap may be used in hypopharyngeal reconstructions as well); the nasolabial options for peri-nasal and lip defects; and the temporal flap for wide defects of the maxillary bone—yielded reliable healing and timely adjuvant therapy while minimizing anesthesia time and monitoring demands. Ultimately, integrating patient comorbidities, prior treatments, defect geometry, functional priorities, and institutional expertise enabled a personalized reconstructive approach in which pedicled flaps complemented, rather than replaced, free tissue transfer—broadening safe, effective options for complex oncologic care.

## Figures and Tables

**Table 1:** Reasons for tissue deficit and surgical interventions (*n*, %).

	*n* (%)
Reasons for tissue deficit	
Not neoplastic	19 (17.0)
Squamous cell carcinoma	80 (71.4)
Adenocarcinoma	3 (2.7)
Adenoid-cystic carcinoma	1 (0.9)
Mucoepidermoid carcinoma	1 (0.9)
Keratoacanthoma	2 (1.8)
Basal cell carcinoma	3 (2.7)
Carcinosarcoma	1 (0.9)
Sarcoma	2 (1.8)
Resective procedures concomitant to flap	
Rhinectomy	3 (2.7)
Glossectomy	34 (30.4)
Maxillectomy	1 (0.9)
Oral cancer removal with mandibulectomy	8 (7.1)
Removal of cheek mucosa tumor	8 (7.1)
Pharyngectomy	3 (2.7)
Pharyngolaryngectomy	5 (4.5)
Parotidectomy extended to the skin	8 (7.1)
Neck dissection extended to the skin	2 (1.8)
Skin tumor removal	24 (21.4)
No resective procedures concomitant to flap	16 (14.3)
Flaps	
PMF	41 (36.6)
DP	20 (17.9)
Platysma	14 (12.5)
PFF	6 (5.4)
TMF	7 (6.3)
Nasolabial	8 (7.1)
SCAIF	7 (6.3)
IHF	2 (1.8)
SCM	1 (0.9)
BFP	4 (3.6)
FAMM	2 (1.8)
Recipient sites	
Nose	11 (9.8)
Tongue	36 (32.1)
Floor of the mouth	9 (8.0)
Cheek mucosa	11 (9.8)
Oropharynx	1 (0.9)
Hypopharynx/cervical esophagus	18 (16.1)
Parotid area	10 (8.9)
Auricular area	4 (3.6)
Superior lip	1 (0.9)
Inferior lip	1 (0.9)
Submental area	3 (2.7)
Lateral neck	2 (1.8)
Maxilla	3 (2.7)
Mandible	2 (1.8)

BFP, buccal fat pad flap; DP, deltopectoral flap; FAMM, facial artery myomucosal flap; IHF, infrahyoid flap; PFF, paramedian forehead flap; PMF, pectoralis major flap; SCAIF, supraclavicular artery island flap; SCM, sternocleidomastoid muscle flap; TMF, temporal muscle flap.

**Table 2:** Complication rates for each pedicled flap (*n*, %).

Flap (*n*)	Partial Necrosis	Complete Necrosis	Wound Dehiscence	Fistula	Bleeding	Pharyngeal Stenosis
PMF (41)	2 (4.9)	1 (2.4)	6 (14.6)	4 (9.8)	1 (2.4)	1 (2.4)
DP (20)	5 (25.0)	1 (5.0)	6 (30.0)	1 (5.0)	2 (10.0)	0 (0.0)
Platysma (14)	2 (14.3)	0 (0.0)	1 (7.1)	1 (7.1)	0 (0.0)	0 (0.0)
PFF (6)	1 (16.7)	0 (0.0)	0 (0.0)	0 (0.0)	0 (0.0)	0 (0.0)
TMF (7)	0 (0.0)	0 (0.0)	1 (14.3)	0 (0.0)	0 (0.0)	0 (0.0)
Nasolabial (8)	0 (0.0)	0 (0.0)	0 (0.0)	0 (0.0)	0 (0.0)	0 (0.0)
SCAIF (7)	2 (28.6)	0 (0.0)	0 (0.0)	1 (14.3)	0 (0.0)	0 (0.0)
IHF (2)	0 (0.0)	0 (0.0)	0 (0.0)	0 (0.0)	0 (0.0)	0 (0.0)
SCM (1)	0 (0.0)	0 (0.0)	0 (0.0)	0 (0.0)	0 (0.0)	0 (0.0)
BFP (4)	1 (25.0)	0 (0.0)	0 (0.0)	0 (0.0)	1 (25.0)	0 (0.0)
FAMM (2)	0 (0.0)	0 (0.0)	0 (0.0)	0 (0.0)	0 (0.0)	0 (0.0)
*p* values	0.370	0.995	0.402	0.960	0.474	0.998

BFP, buccal fat pad flap; DP, deltopectoral flap; FAMM, facial artery myomucosal flap; IHF, infrahyoid flap; PFF, paramedian forehead flap; PMF, pectoralis major flap; SCAIF, supraclavicular artery island flap; SCM, sternocleidomastoid muscle flap; TMF, temporal muscle flap.

## Data Availability

Raw data supporting the conclusions of this article will be made available by the authors on request.

## Abbreviations

The following abbreviations are used in this manuscript:

BFP	Buccal Fat Pad (flap)
DP	Deltopectoral (flap)
FAMM	Facial Artery Myomucosal (flap)
IHF	Infrahyoid Flap
IQR	Interquantile Range
PCF	Pharyngocutaneous Fistula
PFF	Paramedian Forehead Flap
PMF	Pectoralis Major Flap
PMMC	Pectoralis Major Myocutaneous (flap)
PMMF	Pectoralis Major Myofascial (flap)
RFFF	Fadial Forearm Free Flap
SCAIF	Supraclavicular Artery Island
SCM	Sternocleidomastoid (muscle/flap)
SMAS	Superficial Musculoaponeurotic System
SPSS	Statistical Package for the Social Sciences
TL	Total Laryngectomy
TMF	Temporal Muscle Flap
